# Rising levels of antioxidative phyllobilins in stored agricultural produce and their impact on consumer acceptance

**DOI:** 10.1038/s41538-021-00101-7

**Published:** 2021-08-02

**Authors:** Cornelia A. Karg, Christina M. Neubig, Jutta Roosen, Simone Moser

**Affiliations:** 1 Pharmaceutical Biology, Department of Pharmacy, Ludwig-Maximilians University of Munich, Munich, Germany; 2 Technical University of Munich, TUM School of Management, Chair of Marketing and Consumer Research, Freising, Germany

**Keywords:** Molecular biology, Science, technology and society

## Abstract

Consumers often throw away faded greens, because taste and appearance are less appealing compared to fresh ones. We report here a family of antioxidants, the phyllobilins, which increase during storage in iceberg lettuce and cucumber. We show that informing consumers about rising levels of phyllobilins leads to a longer willingness to consume faded lettuce and to an improved health and safety perception.

Currently 11% of the world population suffer from poverty and hunger while one third of the food produced for human consumption worldwide (roughly 1.3 billion tons) is wasted every year^1^. Especially in these times, in a worldwide pandemic, when food systems are stressed and produce accessibility may be limited, finding means to achieve a more sustainable use of food resources is of utmost importance.

In the long term, reducing food waste is crucial for preventing food insecurity in addition to curbing environmental harm, since when food is wasted the resources for its production are wasted as well. Handling and transportation of agricultural produce adds to the total of wasted resources when food is thrown away at the end of the supply chain. The awareness of wasted food has already reached the population, as can be seen by the discussion about the phenomenon of ‘Dumpster Diving’ (salvaging dumped food from large industrial or commercial dumpsters), which is illegal in many countries^2^. While Dumpster Diving can be mainly seen as a consumer demand towards retailers to reduce their food waste, it is important to note that in Europe >50% of food waste is caused by the consumer^3^, stressing the immense potential of changing consumer behavior. Fruits and vegetables are among the food items that are most often wasted by the end consumer^4^. This waste occurs for a variety of reasons including cosmetic issues. Fresh and more chromatic colored foods are often preferred, especially for red and green produce^5^. Consequently, changes in shape or color (e.g. withering or yellowing of lettuce) can influence food practice at home. Consumers often throw away perishable food when it appears to be less fresh, although it is still safe to eat^6^. While there is extensive research concerning preferences regarding how suboptimal looking produce (such as curved cucumbers) may influence food waste, there is little research into how reduced freshness and yellowing of lettuce impact consumer food waste.

With the aim of reducing food waste, what if one could shift consumer behavior towards judging aged produce as still valuable and too good to be thrown away? By informing consumers about a family of antioxidants that occur in food stored for a couple of days, we were able to initiate a revaluing of stored lettuce by consumers.

The loss of green color is the result of a detoxification process employed by plants to get rid of chlorophyll. The biochemical degradation of the green plant pigment chlorophyll takes place in all higher plants, and is triggered by environmental or biotic factors, such as drought or pest infestation^7^, in addition to senescence. At the end of this multi-step program, more water-soluble and stable chlorophyll catabolites called phyllobilins accumulate^7,8^ (Supplementary Fig. 1). Phyllobilins are linear tetrapyrroles and, unlike chlorophyll and its early degradation products such as pheophytin or pheophorbide a^9^, not phototoxic. Recently, phyllobilins were even shown to possess relevant bioactivities: our research revealed strong antioxidative effects in vitro and in cells, even superior to known antioxidants such as bilirubin, Trolox, and caffeic acid. In addition, phyllobilins were shown to be free radical scavengers and to protect cells from oxidative stress^10,11^. Although phyllobilins are ubiquitous in plants, they have not yet been considered as antioxidants in foods, and appear to be completely overlooked components of our nutrition. Phyllobilins are related to phycocyanobilin from the blue-green algae spirulina, which has recently been recognized to have health-promoting effects^12,13^. Consequently, spirulina products have conquered the market and spirulina itself is now considered a ‘superfood’.

Vegetables are major dietary sources of micronutrients (i.e. vitamins and minerals) and bioactive compounds (e.g. carotenoids, flavonoids); the contents of bioactive phytochemicals, however, can vary over time, and many studies focus on the analysis of post-harvest composition of bioactive compounds in plant-based food. Levels of flavonoids, for example, have been found to be stable during storage in spinach^14^; for glucosinolates in broccoli, even an increase was identified during short-time storage^15^; whereas losses of carotenoids and folate contents were reported for packaged spinach^16^. High quantities of phyllobilins have been detected in stored plant-based foods^17,18^, indicating the biochemical chlorophyll degradation program to be active post-harvest.

We selected iceberg lettuce and cucumbers for our studies since they are commonly consumed all over the world. Although lettuce is a source of fiber, vitamins, and folate, amounts in iceberg lettuce were found to be comparably low in regard to other types of lettuce^19^; in general, lettuce and cucumber are lower in nutrients than some other greens^20,21^. In contrast, high amounts of phyllobilins were detected in these two vegetables in a preliminary screening of a variety of plant-based foods. Using time-lapse photography, we monitored the aging process of iceberg lettuce and cucumbers during a storage time of 7 and 12 days, respectively, until the food appeared faded but still edible (Fig. 1a). To ensure that aged lettuce and cucumbers were still safe to consume, we confirmed a lack of microbial increase at the end of storage. Furthermore, isolated phyllobilins were tested for chemo- and genotoxicity and were found to be innocuous (Supplementary Figs. 2, 3, and 4).

**Fig. 1:**
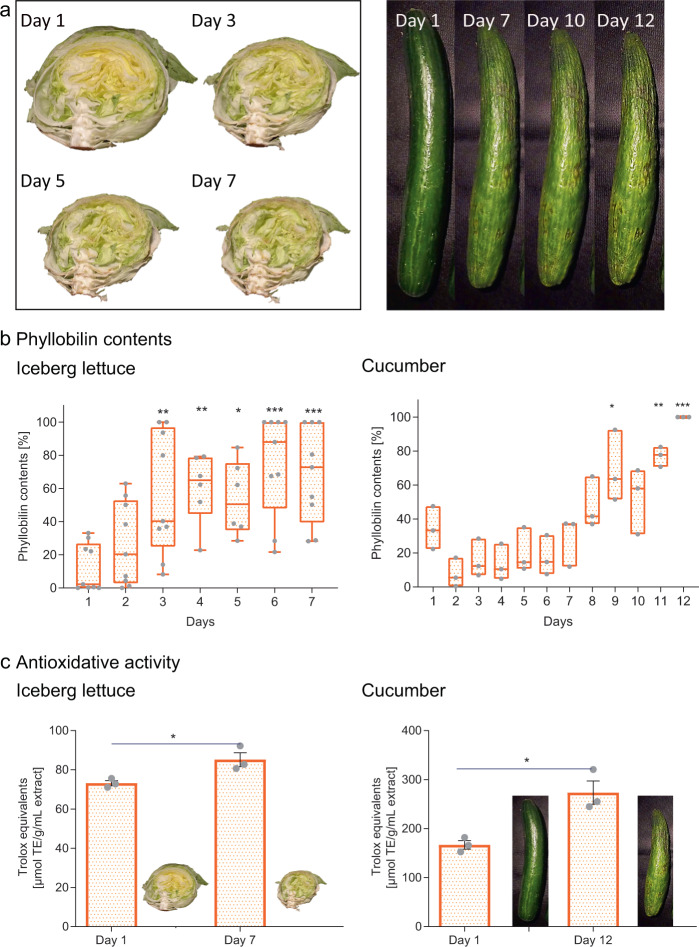
Analyses of stored greens. **a** Snapshots of a time-lapse study (videos available as Supplementary Material) showing the aging progress of iceberg lettuce and cucumbers during home storage. **b** Phyllobilin contents in lettuce and cucumbers significantly increased with storage time (box-and-whiskers plot (lettuce): box 25–75%, whiskers min–max, line at median; floating bars (cucumber): min–max, line at mean value; *p < 0.05, **p < 0.01, ***p < 0.001). **c** End-of-storage extracts of the lettuce and cucumbers have significantly higher antioxidative in vitro activity compared to freshly bought produce. Values expressed relative to the antioxidant Trolox (*p < 0.05). Mean values ± s.e.m are shown.

Each day, samples of lettuce and cucumber peels were collected and phyllobilin contents were analyzed (Supplementary Fig. 5). On day 1, when the lettuce and cucumber still appeared fresh and green (Supplementary Fig. 6), only low levels of phyllobilins were detected; the amount of phyllobilins, however, increased significantly over time. For lettuce, phyllobilin content increased significantly throughout days 3 through 7 compared to day 1. For cucumbers, phyllobilin content started to increase significantly on day 9 and reached its maximum level on day 12 (Fig. 1b). Next, we compared the antioxidant potential of lettuce and cucumber extracts at the beginning and end of storage. Although phyllobilins are formed at the expense of chlorophyll, which is connected to biological activities^22^, extracts of aged produce enriched in phyllobilins showed significantly higher antioxidative activity compared to extracts of fresh ones (Fig. 1c). Furthermore, we performed targeted cellular biological assays using phyllobilins isolated from aged lettuce and cucumber, which demonstrated a similar or higher antioxidative potential than Trolox and revealed radical scavenging activities, protecting cells upon oxidative stress (Supplementary Fig. 7).

Phyllobilins are therefore potent antioxidants that could—depending on storage time—constitute a noteworthy part of human nutrition. Phyllobilin levels rise from 0.07 to 2.77 µg/cm^2^ in lettuce and from 1.34 to 4.52 µg/cm^2^ in cucumber, as calculated from the data of the time-lapse study (Supplementary Fig. 8). We investigated whether educating consumers about strong antioxidative components that occur nearly exclusively in aged produce has the potential to shift consumer behavior towards eating stored produce longer, which holds a large promise of preventing unnecessary food waste.

Using an online experiment, we show that consumers who received information about health benefits and occurrence of phyllobilins in aged iceberg lettuce (‘phyllobilin condition’) were willing to eat the lettuce significantly longer (by 0.56 days, *p* = 0.001, Supplementary Table 3) than consumers who received a non-relevant information about chlorophyll (‘control condition’). While similar in the beginning, willingness to consume the lettuce dropped quickly in the control condition after day 3, while decreasing significantly more slowly in the phyllobilin condition (Fig. 2a). Moreover, consumers in the phyllobilin condition rated the lettuce significantly healthier and safer than those in the control condition on days 4–7 (Fig. 2b, c). Compared to a safety condition, in which consumers were informed that 7 days old lettuce is still safe to eat, the phyllobilin condition rated the lettuce significantly healthier on days 6 and 7; and even though the lettuce’s safety was emphasized in the information given to subjects in the safety condition, consumers in the phyllobilin condition perceived the lettuce as significantly more safe on day 6 (Supplementary Tables 4 and 5). Interestingly, an increased health perception in the phyllobilin group compared to the safety group led to a comparable behavior, as reflected in similar results for the willingness to consume the stored lettuce between the two groups. Considering this fact, the highest impact on food waste might be achieved by combining safety and health, and educate people that aged, stored lettuce is not only safe to eat but has even higher antioxidative potential than fresh lettuce.

**Fig. 2:**
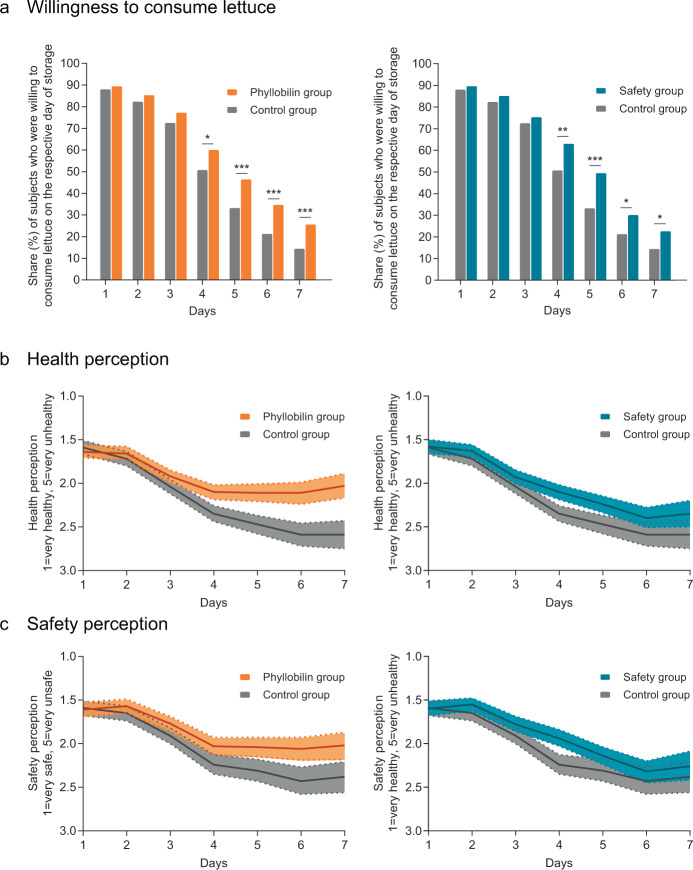
Results of consumer online experiment (*N* = 1103). **a** Share (%) of subjects in the phyllobilin and control condition (left panel) and safety and control condition (right panel) who stated to be willing to consume the lettuce on days 1–7 of storage (bars, *p < 0.05, **p < 0.01, ***p < 0.001, statistical analysis see Supplementary Table 3). **b** Health and **c** safety perception of the lettuce on a 5-point Likert scale on days 1–7 (mean values and 95% CI, statistical evaluation see Supplementary Tables 4 and 5).

The knowledge about phyllobilins has the potential to upgrade longer stored greens, and initiate a rethinking in consumers. Our results indicate that informing consumers about phyllobilins can change their health and safety perception of produce and leads to a longer willingness to eat perishable produce (even though they may be wrinkly or yellow instead of fresh and green), which has great potential to reduce food waste at home. In Germany, for instance, roughly 188.000 tons of lettuce are consumed per year^23^. Regarding this high number, prolonging willingness to consume lettuce by only an average of half a day (as indicated by our results) will already make a substantial contribution to reducing food waste. Phyllobilins are ubiquitous in plants and have been overlooked as food components for far too long: with this report and its implications in consumer behavior, we aim at raising awareness of these compounds in the food science community – we hope to initiate in-depth evaluations of phyllobilins in terms of animal and human studies, which will be necessary to substantiate their use in health claims and dietary recommendations. Eventually, educating consumers about this class of powerful antioxidants can lead to a more sustainable consumer behavior and hence has a huge potential for reducing consumer food waste at home.

## Materials and methods

### Reagents and chemicals

HPLC grade acetonitrile (MeCN), methanol (MeOH), ethanol (EtOH), dimethylsulfoxide (DMSO), hydrochloric acid (HCl), and filter paper (110 mm pore size) were obtained from VWR International GmbH (Ismaning, Germany), and ultra-pure water (18MΩ.cm^−1^) from a Millipore S.A.S. Milli-Q Academic system (18,2 MΩ cm^−1^, Molsheim, France); 2,4,6-Tri(2-pyridyl)-s-triazine (TPTZ), potassium phosphate monobasic (KH_2_PO_4_), potassium phosphate dibasic (K_2_HPO_4_), ammonium acetate (NH_4_AcO), iron(III) chloride (FeCl_3_), hydrogen peroxide (30%) were from Merck KGaA (Darmstadt, Germany). Yeast extract peptone and MEM non-essential amino acid solution were obtained from Sigma-Aldrich (Taufkirchen, Germany) and collagen G from Biochrom AG (Berlin, Germany). Trolox was from Enzo Life Sciences GmbH (Lörrach, Germany), 2′,7′-dichlorodihydrofluorescein diacetate (H_2_DCF-DA) and LB Broth Base were from Thermo Fisher (Waltham, MA, USA). DMEM medium was from PAN-Biotech GmbH (Aidenbach, Germany); MEM medium was from PromoCell (Heidelberg, Germany) and HEPES from AppliChem GmbH (Darmstadt, Germany). Fetal calf serum (FCS) was purchased from PAA Laboratories GmbH (Pasching, Austria) and l-glutamine, agar, and glucose were obtained from Carl Roth (Karlsruhe, Germany). Iceberg lettuce and cucumber were bought at a local supermarket and immediately used.

### Chromatography

(i) Analytical HPLC: Agilent 1260 Infinity II LC system with a 1260 Infinity Degasser, a 1100 Series quaternary pump and 1100 Series diode array detector; column: Phenomenex Hyperclone (ODS 5 µm, 250 × 4.6 mm i.d.) at 15 °C protected by Phenomenex ODS 4 × 3 mm i.d. pre-column; injection volume: 100 µL, flow rate 0.5 mL/min. Solvent system: mobile phase *A* = 10 mM ammonium acetate in water, *B* = MeCN; Solvent composition *A*/*B* (v/v): 0–5 min: 95/5, 5–45 min: 95/5 to 40/60, 45–51 min: 40/60 to 0/100, 51–53 min: 0/100, 53–55 min: 0/100 to 95/5. Data were collected and processed using OpenLab CDS Data Analysis 2.3.
(ii) Semi-preparative HPLC: Gynkotek LC-System with manual sampler, M480 pump, Phenomenex DG-301 online degasser, Gynkotek UVD 640 diode array detector and a Rheodyne injection valve with a 5-mL loop; column: Phenomenex Luna 5 µm C18, 100 Å protected by Phenomenex pre-column ODS 9 × 16 mm, flow rate 2.5 mL/min. Solvent system: mobile phase *A* = 10 mM ammonium acetate in water, *B* = MeCN; solvent composition *A*/*B* (v/v): 0–2 min: 88/12, 2–12 min: 88/12 to 80/20, 12–30 min: 80/20 to 20/80, 30–40 min: 20/80 to 0/100. Data were collected and processed using Gynkosoft 5.50.
(iii) Analytical HPLC LC/MS: Shimadzu HPLC system equipped with an LC-20AD pump, a DGU-20A5 online degasser unit and an SPD-M20A diode array detector, a Rheodyne injection valve with a 200-µl loop; column: Phenomenex Hyperclone (ODS 5 µm 250 × 4.6 mm i.d.), protected by Phenomenex ODS 4 × 3 mm i.d. pre-column; flow rate 0.5 mL/min. Solvent system: mobile phase A = 4 mM ammonium acetate in water, *B* = 4 mM ammonium acetate in MeOH; solvent composition *A*/*B* (v/v): 0–5 min: 80/20; 5–60 min: 80/20 to 0/100; 60–70 min: 0/100; 70–75 min: 0/100 to 80/20. Data were collected and processed using Shimadzu LC Solution software (version 1.24 SP1).

### Spectroscopy

UV/Vis: λ_max_ [nm] (εrel), Thermo Spectronic Genesys 5 (336001) UV-Visible spectrophotometer. Concentrations of the phyllobilins were calculated using log *ε* (312 nm) = 4.23 for the phylloleucobilin (PleB)^24^, log *ε* (426 nm) = 4.51 for the phylloxanthobilin (PxB)^25^, and log *ε* (237 nm) = 4.49 for the iceberg lettuce fraction^26^. High resolution (HR) mass spectrometry (MS) (LC-HR MS system): ThermoScientific QExactive Orbitrap mass spectrometer, equipped with an ESI source (positive-ion mode, spray voltage 3.7 kV. Data were collected and processed with Xcalibur 4.1 software.

### Time-lapse photography

To record the aging process of stored iceberg lettuce and cucumbers, iceberg lettuce were stored for 7 days and cucumbers for 12 days at 22 °C, 26% RH, and protected from light, until the plant produce appeared wrinkly and de-greened but still edible. Pictures were taken during storage every 90 min with a fixedly installed GoPro HERO4 camera. Data were processed using ImageJ 1.45 s.

### Determination of phyllobilin contents

Each day during the storage time of 7 days for iceberg lettuce and 12 days for cucumber, samples of the same size of lettuce leaves and cucumber peels were collected and stored at −20 °C until further use. Before analysis, samples were thawed, cut into halves, and one part was used for the analysis of the phyllobilin contents. Photos of the specimens were taken with a Canon DS126181 camera and the areas of the samples were determined by using Matlab software R2018b. The samples were ground with 700 µL of MeOH/potassium phosphate buffer 100 mM pH 7 (PBS) (20/80) in a mortar and centrifuged several times at 14,000 rpm for 4 min, before an aliquot of 120 µL was analyzed by analytical HPLC. Phyllobilins were tentatively identified by their characteristic UV Vis online spectra^8^ using a diode array detector and integrated with the Agilent software OpenLab CDS; further, identified phyllobilins were confirmed by mass spectrometry (MS) and liquid-chromatography-high resolution mass-spectrometry (LC-HR MS) (Supplementary Figs. 9 and 10 and Supplementary Table 1). Peak areas of all detected phyllobilins were summarized and corrected for the differences of the area of each sample. A correction factor, which accounts for the loss of volume due to dehydration effects, was determined by analyzing the area of lettuce and cucumbers on the snap-shots of the time-lapse images by Matlab software R2018b. The values were multiplied with the calculated factor and normalized to the sum of all phyllobilins at the day with the highest phyllobilin amount. For iceberg lettuce three series with three lettuce each were performed; samples of one of the series were collected on days 1, 2, 3, 6, and 7. Three cucumbers were analyzed each day during storage.

### Extraction of iceberg lettuce and cucumbers

Extracts of three iceberg lettuce and cucumbers at day 1, when they appeared fresh and green, and at the last day of storage (7 and 12, respectively) at a more aged and de-greened state, were prepared as follows: iceberg lettuce (without the stem) were weighed and crushed with a Kenwood CH580 Food Processor. The slurry was then extracted with 500 mL of ethanol and again mixed with a Braun hand blender Model MR 5000. The mixture was first filtered through a coffee filter and then again filtered through a filter paper (110 mm pore size). The residue was washed with 200 mL of ethanol and again filtered. Cucumbers were completely peeled; the peel was weighed and crushed with a Braun hand blender Model MR 5000 with 100 mL of ethanol. The mixture was filtered through a filter paper (110 mm pore size) and the residue was washed with 100 mL of ethanol. Extracts of iceberg lettuce and cucumber were stored at −20 °C and used for determination of the in vitro antioxidative activity.

### Isolation of phyllobilins from stored iceberg lettuce and cucumber

Iceberg lettuce and cucumbers used for the time-lapse photography studies were extracted after 7 and 12 days of monitoring the aging process. The lettuce and cucumber peels were crushed with a Kenwood CH580 Food Processor, mixed with 500 mL of ethanol each, and again crushed with a Braun hand blender Model MR 5000. The extracts were filtered through a coffee filter and dried on a rotary evaporator. The residue was re-dissolved in 20/80 MeOH/ PBS 100 mM pH 7 and centrifuged prior to purification by semi-preparative HPLC using a diode array detector. A phyllobilin-enriched fraction was isolated from the extract of iceberg lettuce (LF) and two phyllobilins, a PleB and a PxB, were isolated from the peels of cucumber. The purity of the PleB, the PxB, and the LF was confirmed by analytical HPLC. The samples were lyophilized, dissolved in DMSO and stored at −20 °C until further use. The concentrations were determined spectrophotometrically by using the extinction factors as described above.

### Cell culture

Human embryonic kidney derived HEK293 cells were obtained from the German Collection of Microorganisms and Cell Cultures (DSMZ) and Caco-2 cells were a kind gift of Prof. Wagner from the Chair of Pharmaceutical Biology and Biotechnology at the LMU Munich. HEK293 cells were cultured in DMEM Medium with 10% fetal calf serum (FCS) and Caco-2 cells in MEM Medium with 10% FCS, 1% non-essential amino acids (NEAA), 1 M HEPES, and 1% glutamine. All cells were cultivated at a constant humidity with 5% CO_2_ at 37 °C. The culture flasks and multiwell plates for HEK293 cells were coated with collagen G (0.001% in PBS).

### Ferric Reducing Antioxidant Power assay

The Ferric Reducing Antioxidant Power (FRAP) assay allows for the determination of the antioxidant capacity of biological samples in vitro and was performed according to the protocol of Benzie et al.^27^ with minor adaptations. Briefly, FRAP reagent was prepared freshly by combining 10 volumes of 300 mM acetate buffer pH 3.5, 1 volume of 10 mM TPTZ (2,4,6-Tri(2-pyridyl)-s-triazine) in 40 mM HCl, and 1 volume of 20 mM iron(III)chloride. The prepared extracts or isolated compounds and varying concentrations of Trolox were incubated with FRAP reagent for 5 min at 37 °C. The reduction of the Fe^3+^-(TPTZ)_2_-complex by a possible antioxidative activity is measured colorimetrically with a Tecan SpectraFluor plus microplate reader at 620 nm. A calibration curve with different Trolox concentrations was calculated and antioxidant potential of the extracts and isolated compounds was expressed as Trolox equivalents (µmol TE/µM or µmol TE/g/mL extract).

### Detection of intracellular reactive oxygen species

The antioxidative activity of isolated phyllobilins in cells was determined as described^28^ with minor modifications: In brief, cells were seeded in 96-well plates and allowed to adhere for 24 h. Then, diluted compounds (PleB 10, 20, 50 µM; PxB 10, 20 µM; and LF 20, 50 µM) and a vehicle control were added to the cells and incubated for 24 h. Medium was discarded and cells were treated with H_2_DCF-DA (10 µM) for 30 min, before washing cells with PBS + Ca^2+^+ Mg^2+^ and adding hydrogen peroxide (1 mM) for 30 min. Cells treated with a vehicle control and without H_2_O_2_ served as negative control. The fluorescence of oxidized H_2_DCF-DA, the highly fluorescent 2′,7′-dichlorofluorescein (DCF), was detected with a Tecan SpectraFluor plus microplate reader (excitation wavelength 485 nm; emission wavelength 530 nm). Data were normalized to the positive control, which was treated with hydrogen peroxide and a vehicle control.

### Cell viability assay

Isolated phyllobilins were probed for their toxicity in cells. Thus, cell viability of treated HEK293 and Caco-2 cells was determined by a CellTiter-Blue® assay (Promega). The assay allows to determine cell viability as viable cells are able to reduce the dye resazurin into the fluorescent reagent resorufin and nonviable cells show reduced metabolic capacity, which results in a reduced or no fluorescence signal. Cells were treated as described above with isolated compounds for 24 h. In all, 4 h before the end of stimulation time, CellTiter-Blue® reagent was added and after 4 h of incubation at 37 °C, fluorescence of resorufin at 590 nm was measured with a Tecan SpectraFluor plus microplate reader. The number of viable cells was normalized to a DMSO control.

### Alkaline comet assay

Oxidative and environmental stress in cells often leads to degradation and oxidation of genomic DNA. The alkaline comet assay was performed as a biomarker to confirm that isolated phyllobilins cause no genotoxic effects. A commercial Comet SCGE assay kit from Enzo Life Sciences GmbH (Lörrach, Germany) was used, which detects DNA damage by determination of the integrity of DNA release from cells immobilized in agarose. When DNA gets fragmented, the small parts move more easily in the gel than undamaged DNA, which creates a characteristic “comet” shaped tail. With a specific dye, nucleic acid can be stained and tail formation can be analyzed by CometScore software and compared to controls. Cells were treated with PxB (20 µM), PleB (50 µM), LF (50 µM), and a vehicle control for 24 h. H_2_O_2_ treated cells (4 °C, 20 min) served as a positive control. Cells were then seeded in low melting agarose at 37 °C on pre-coated glass slides and lysed overnight in lysis buffer at 4 °C. Slides were incubated in alkaline solution and electrophoresis was performed at 35 V, 300 mA for 30 min in alkaline electrophoresis buffer. Glass slides were washed in water, fixed in 70% ethanol for 5 min, and completely dried. DNA was then stained with CYGREEN® Nucleic acid dye for 30 min in the dark before slides were washed and again dried at 37 °C. Images were taken with a Leica TCS SP8 SMD microscope (Leica Microsystem, Wetzlar, Germany). Additionally, tail moments (% DNA in tail × tail length) of 20 cells per condition were analyzed with CometScore.

### Microbial load

The microbial load of iceberg lettuce and cucumber was determined to confirm that even after 7 days or 12 days of storage, the plant produce showed no increased microbial contamination and could still be safely consumed. Three iceberg lettuce and cucumbers were bought at a local supermarket and microbial load was assessed at days 1 and 7 for the lettuce and at days 1 and 12 for the cucumber as follows: vegetables were washed thoroughly. A small piece of the outer leaf of each lettuce and a small slice of each cucumber was weighed under sterile conditions. Each sample was soaked in 15 mL of sterile water for 30 min and the solution was diluted 1:100 or 1:10 before three aliquots of 100 µL of each sample were inoculated on plates with LB medium (2% LB broth base, 3.2% agar) and Yeast Extract–Peptone–Dextrose (YPD) medium (1% yeast extract, 2% peptone, 2% agar, 20% glucose). LB medium was used to cultivate bacteria and YPD medium to cultivate fungi. Plates were incubated at 29 °C for 24 h for lettuce and 68 h for cucumber, colonies were counted, and Colony Forming Units (CFU) per mL, corrected for the weight of the sample, were calculated.

### Statistics

All experiments were performed three times in triplicates unless stated otherwise. The data represent means ± s.e.m. Statistical differences were assessed by using an unpaired *t*-test or a one-way analysis of variance with post hoc analysis using Dunnett’s multiple comparison test. Statistical analyses were carried out with GraphPad Prism software version 7.05.

### Consumer behavior study

The study was carried out in accordance to the Technical University of Munich guidelines for good scientific practice. Data were collected in July 2020 through an online experiment with a between-subjects design in Germany (*N* = 1103, Supplementary Table 2). The sample was quota-sampled to ensure representativeness for the German population regarding age, gender, region, and education. Informed consent was obtained from all participants. Subjects were randomly assigned to three different treatment groups (control, safety, phyllobilin). First, subjects provided demographical information and answered questions about food consumption and habits. Next, all groups were shown the same time-lapse video of an iceberg lettuce head aging for 7 days, but received different information with the video. The safety group (*N* = 376) learned that even 7-days-old iceberg lettuce is still safe to eat. The phyllobilin group (*N* = 360) learned about phyllobilins, their health benefits, and occurrence in aged iceberg lettuce. The control group (*N* = 367) was given information that was not expected to change their perception and willingness to consume the lettuce (i.e., they learned about chlorophylls and photosynthesis). In order to ensure that subjects had read the information, they were asked to answer three knowledge questions. In the final part of the questionnaire, subjects were shown up to seven pictures of the aging iceberg lettuce taken on consecutive days. For each picture and day, they indicated on a scale from 1 to 5 how healthy and how safe to eat they judged the respective lettuce (1 = very healthy/safe; 5 = very unhealthy/unsafe). Moreover, they indicated whether they would still eat the lettuce (yes/no) (Supplementary Table 3 and Supplementary Fig. 11). Data were analyzed in IBM SPSS Statistics 26 using descriptive statistics and One-way Analyses of Variance. Due to the large sample size, normal distribution of variables was assumed.

## Supplementary information

Supplementary Information

Supplementary Movie 1

Supplementary Movie 2

## Data Availability

The datasets generated during and/or analyzed during the current study are available from the corresponding author on reasonable request.
