# Supplementary material for: Rising levels of antioxidative phyllobilins in stored agricultural produce and their impact on consumer acceptance
Source: NPJ Sci Food. 2021 Aug 2;5:19. doi: 10.1038/s41538-021-00101-7 (PMC8329175; doi:10.1038/s41538-021-00101-7)
Supplement: Supplementary file 1 — Supplementary Information [file 41538_2021_101_MOESM1_ESM.pdf]

Rising levels of antioxidative phyllobilins in stored agricultural produce and their impact on consumer acceptance

Cornelia A. Karg[a], Christina M. Neubig[b], Jutta Roosen[b], Simone Moser[a,*]

[a]Pharmaceutical Biology, Department Pharmacy, Ludwig-Maximilians University of Munich, Butenandtstraße 5-13, D-81377 Munich, Germany

[b] Technical University of Munich, TUM School of Management, Chair of Marketing and Consumer Research, Alte Akademie 16, D-85354 Freising, Germany

*Email: simone.moser@cup.uni-muenchen.de

Supplementary Information

Phyllobilins (PBs) are linear tetrapyrroles, which arise from the degradation of the green plant pigment chlorophyll. Scheme 1 shows the most important phyllobilin core structures, which are generated during chlorophyll degradation: a phylloleucobilin (PleB) or non-fluorescent chlorophyll catabolite (NCC), which was the first elucidated structure of chlorophyll catabolites and was found to accumulate in the vacuoles of the plant cell; a phylloxanthobilin (PxB) or yellow chlorophyll catabolite (YCC), first characterized in 2008 and representing an oxidation product of the PleB; and a dioxobilin-type PleB (DPleB), resulting from another branch of chlorophyll breakdown and carrying a lactam group instead of an aldehyde moiety at the north-eastern hemisphere[1].

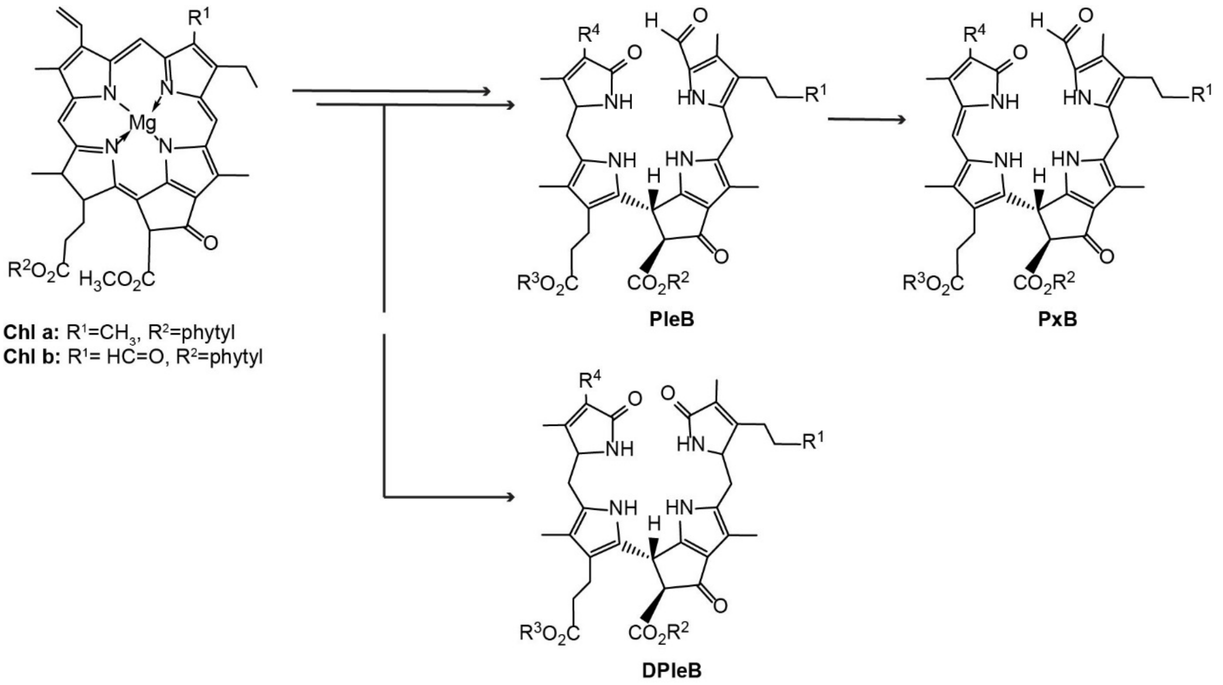

Supplementary Figure 1: Overview of phyllobilin key structures formed during chlorophyll breakdown.

Since the first structure of a PB was elucidated in 1991, more than 30 constitutionally different structures were identified, which, however, all share the same tetrapyrrolic core pattern. The extensive characterization of PBs by mass spectrometry led to the creation of a MS database and mass spectrometry was established as tool for the structural elucidation of PBs with known modification patterns[2,3]. Therefore, phyllobilins in extracts of iceberg lettuce and cucumber were identified by liquid-chromatography-high resolution mass-spectrometry (LC-HR MS), due to characteristic UV Vis spectra and known mass patterns from the library.

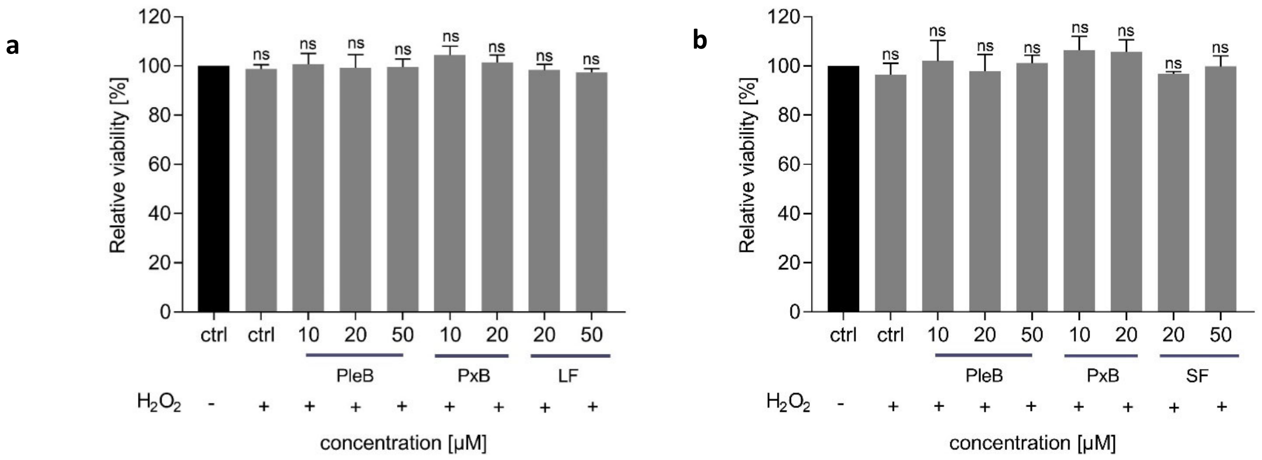

Supplementary Figure 2. Isolated phyllobilins do not alter cell viability at indicated concentrations. Cell viability was determined by a CellTiter-Blue® assay in HEK293 (**a**) and Caco-2 (**b**) cells. Values represent mean ± SEM of three independent experiments. One-Way ANOVA followed by Dunnett's multiple comparison test, ns = not significant.

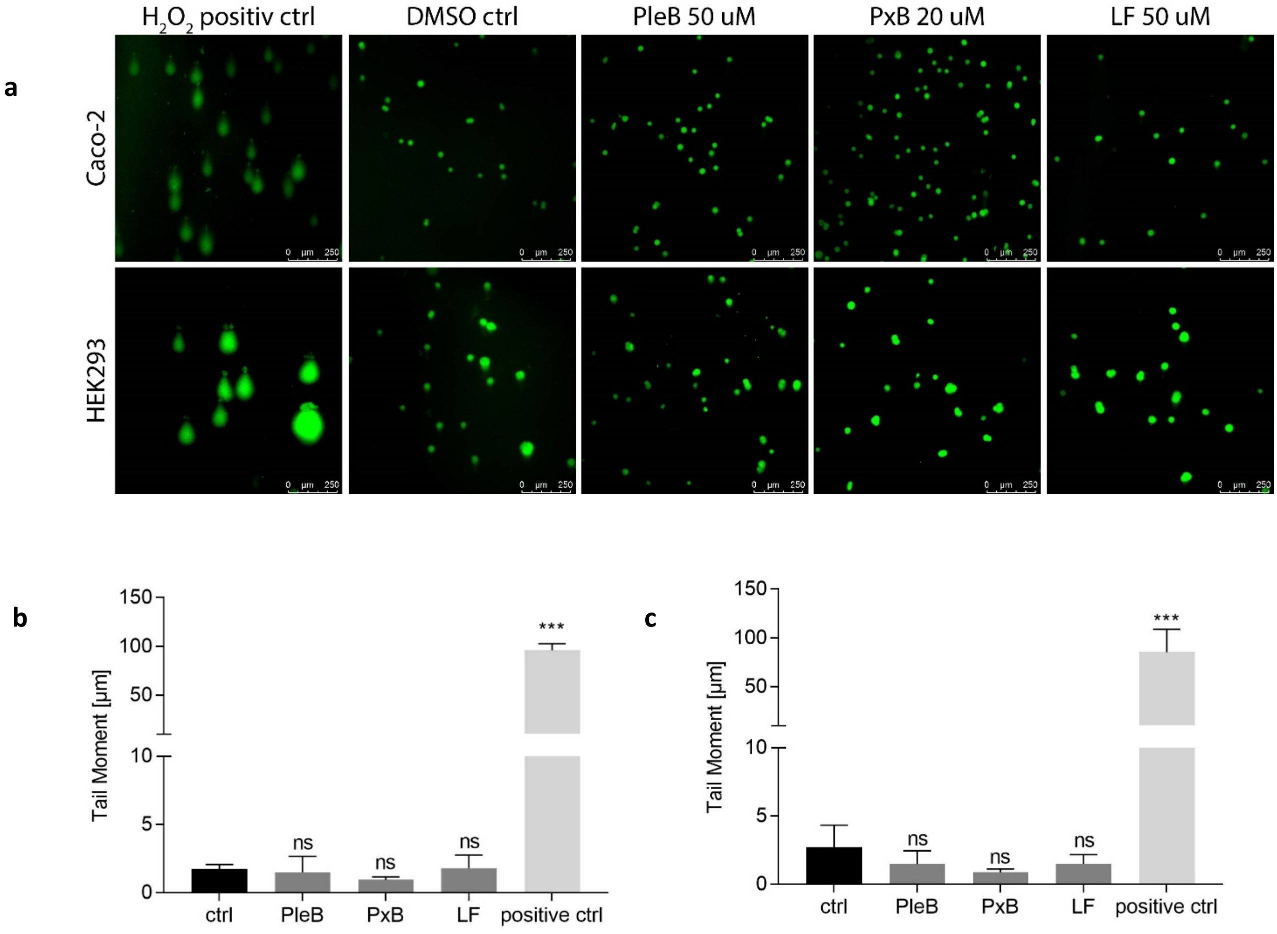

Supplementary Figure 3: Isolated phyllobilins show no DNA damaging effects. Isolated PleB (50 µM) and PxB (20 µM) from cucumber peels and a phyllobilin enriched fraction (LF) (50 µM) from iceberg lettuce were tested for a possible genotoxic effect using an alkaline comet assay in HEK293 and Caco-2 cells. In contrast to the positive control, which was treated with

hydrogen peroxide for 20 min at 4 °C, isolated PBs showed no effect on DNA fragmentation. Three independent experiments were performed (one representative image is shown, **a**). Additionally, tail moments (% DNA in tail * tail length) of 20 cells per condition were analyzed with CometScore in HEK293 (**b**) and Caco-2 (**c**) cells. One-Way ANOVA followed by Dunnett's multiple comparison test, ***p < 0.001, ns = not significant.

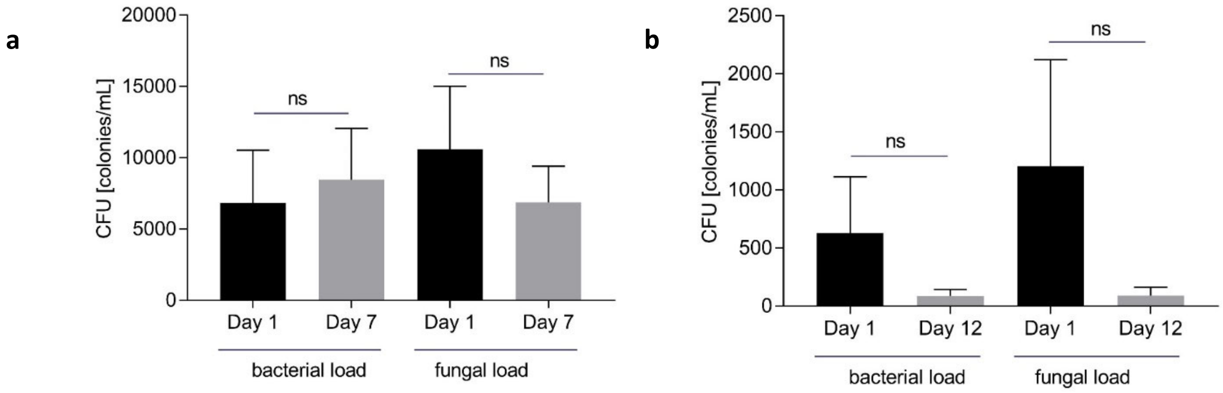

Supplementary Figure 4: Stored iceberg lettuce and cucumber show no increased microbial load. A piece of iceberg lettuce (**a**) and cucumber (**b**) at day 1 and at the last day of storage time was soaked in sterile water for 30 min. The solution was inoculated on agar plates for bacterial growth and fungal growth and incubated for 24 h (iceberg lettuce) and 68 h (cucumber) at 29 °C. Colonies were counted and colony-forming units (CFU) per mL were calculated and corrected for the weight of the respective sample (Unpaired t-test, ns=not significant).

a

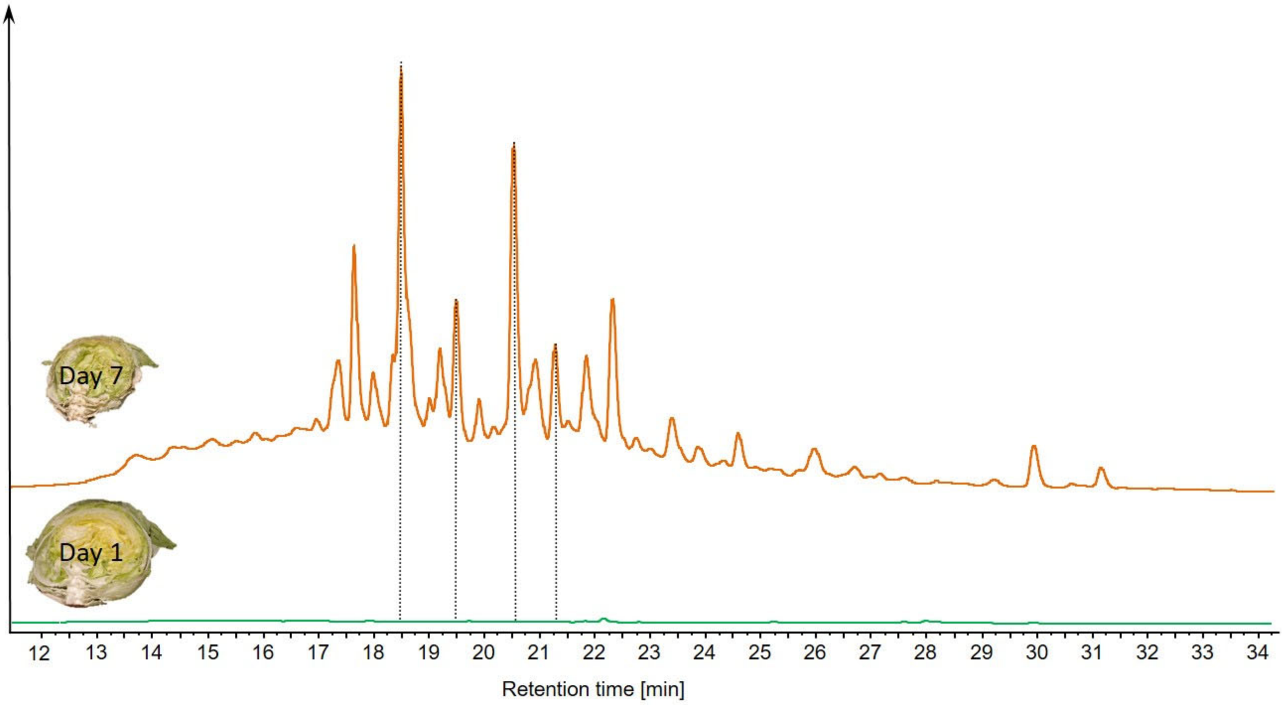
Day 7
Day 1
12 13 14 15 16 17 18 19 20 21 22 23 24 25 26 27 28 29 30 31 32 33 34
Retention time [min]

b

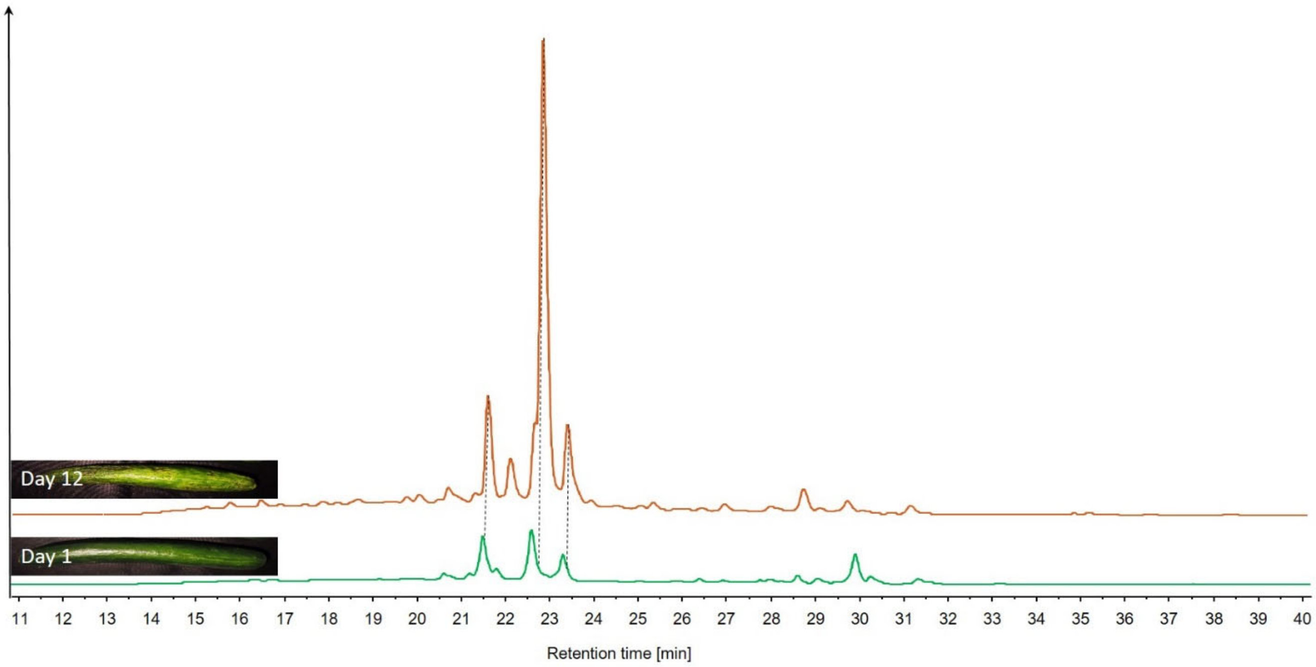
Day 12
Day 1
11 12 13 14 15 16 17 18 19 20 21 22 23 24 25 26 27 28 29 30 31 32 33 34 35 36 37 38 39 40
Retention time [min]

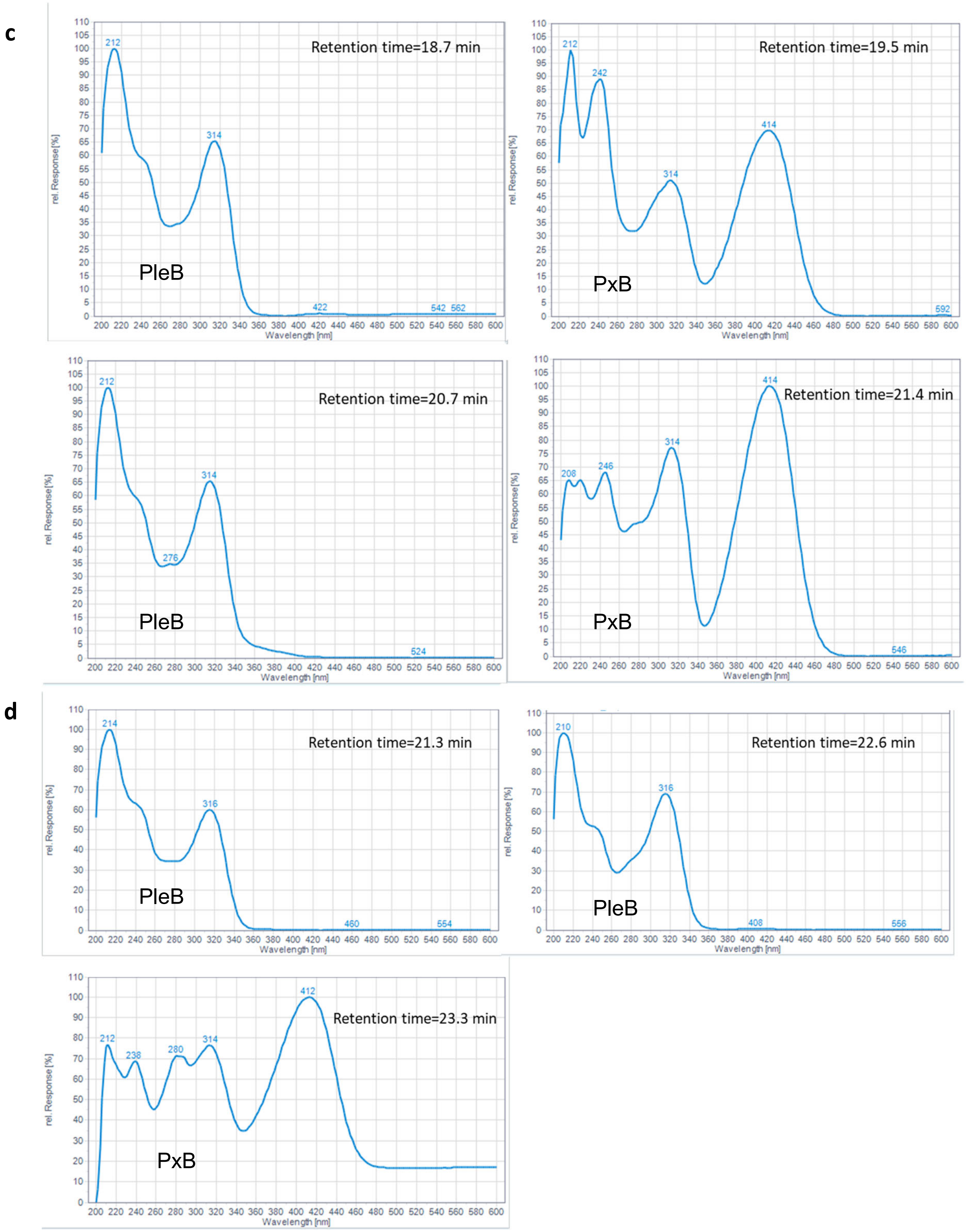

Supplementary Figure 5: Phyllobilin content increases with storage time and progressive age of iceberg lettuce and cucumber. Analytical HPLC traces of extracts of iceberg lettuce (**a**) of day 1 and day 7 and cucumber peels (**b**) of day 1 and day 12 and UV Vis online spectra of identified phyllobilins for iceberg lettuce (**c**) and cucumber (**d**). Iceberg lettuce and cucumber were stored at room temperature and light protected until a progressed aging of the vegetables, although the plant produce still appeared edible. Phyllobilins were tentatively identified by their characteristic UV Vis online spectrum[1] using a diode array detector and

confirmed by LC-HR MS; phyllobilin signals are highlighted with dashed lines. Chromatograms show detection at 320 nm.

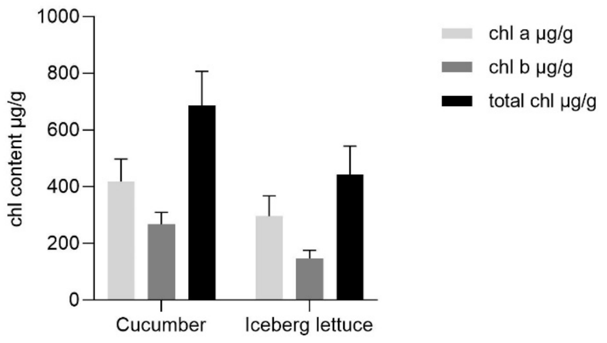

Supplementary Figure 6: Chlorophyll a, chlorophyll b, and total chlorophyll contents in freshly bought cucumber and iceberg lettuce. Samples of cucumber peel and iceberg lettuce of equal weight were extracted four times with 1 ml of 80% acetone containing 1mM KOH[4]. Extracts were combined and chlorophyll content was determined spectrophotometrically as described[5]. For a rough estimation of the chlorophyll content, experiment was performed in triplicates of one specimen each.

a

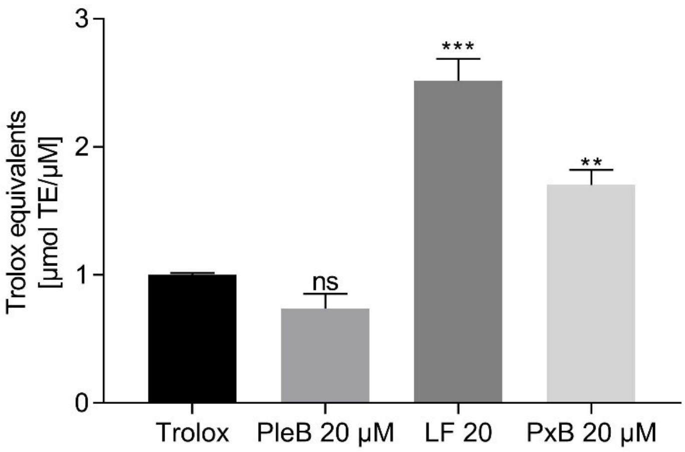

b

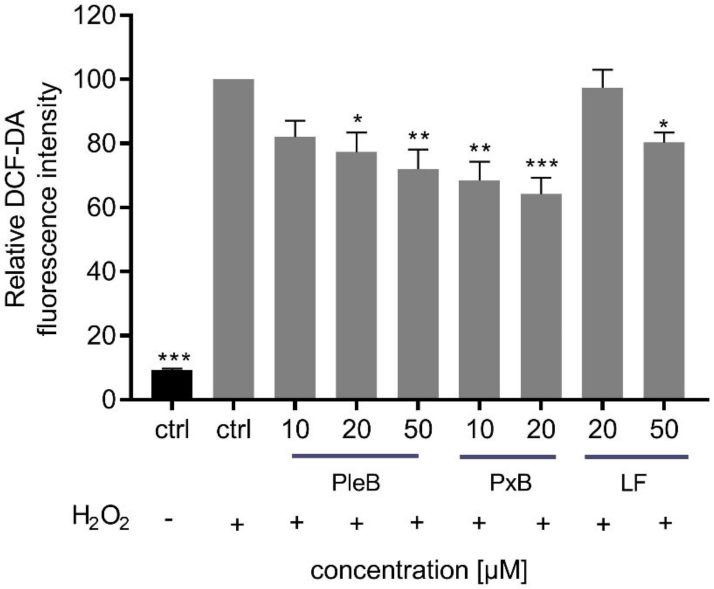

Supplementary Figure 7: Phyllobilins possess antioxidative activities. (**a**) *In vitro* antioxidative potential of a phylloleucobilin (PleB) and a phylloxanthobilin (PxB), isolated from de-greened cucumber peels as described in the materials section, and a phyllobilin-enriched fraction (LF) isolated from aged iceberg lettuce (LF). Antioxidant power was determined by FRAP assay with Trolox as a control and expressed as Trolox equivalents (μmol TE/μM). Phyllobilins showed similar or higher antioxidative activity as the vitamin E derivative Trolox, known for its strong antioxidative effects. One-Way ANOVA followed by Dunnett's multiple comparison test, $^{**}p < 0.01$, $^{***}p < 0.001$, ns = not significant. (**b**) Phyllobilins show *in cellulo* antioxidative activity by scavenging intracellular oxidative stress in a dose dependent manner. HEK293 cells were incubated with PleB, PxB or LF at indicated concentrations for 24 h before stimulating with 1 mM H_2O_2. ROS scavenging activity was determined by the prevented oxidation of the intracellular fluorescein derivative H_2DCF into highly fluorescent DCF. One-Way ANOVA followed by Dunnett's multiple comparison test, $^{*}p < 0.05$, $^{**}p < 0.01$, $^{***}p < 0.001$.

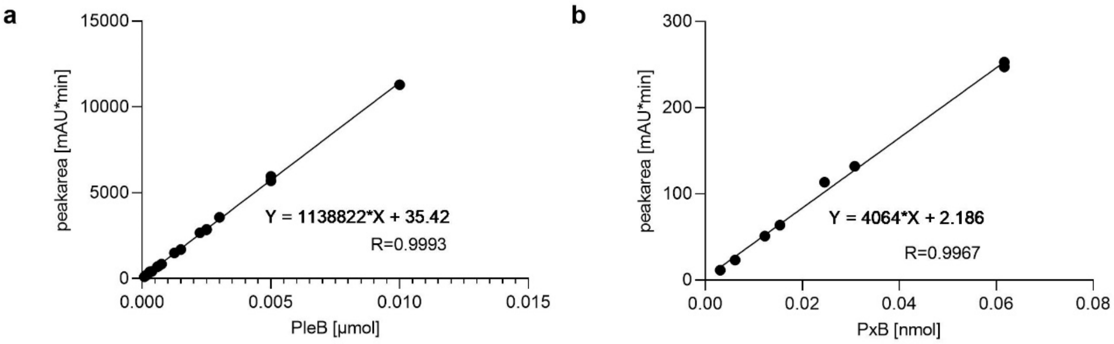

Supplementary Figure 8: Calibration linear of PleB (A) and PxB (B). Different concentrations of PleB and PxB were analyzed by analytical HPLC to fit a calibration linear of PleB and PxB, which were used to estimate the maximum phyllobilin content in cucumber peels and iceberg lettuce after storage, respectively.

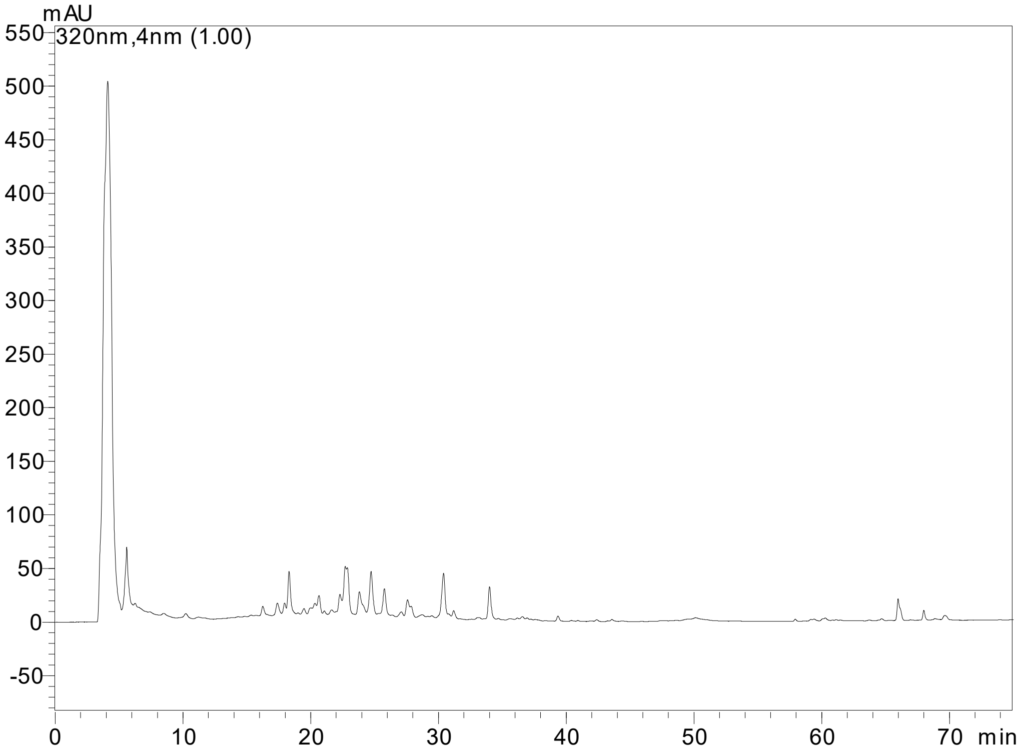

Supplementary Figure 9: HPL-chromatogram of iceberg lettuce recorded during LC-HR MS analysis. Lettuce was stored for seven days in the dark and extracted as described in the methods section. (Detection at 320 nm)

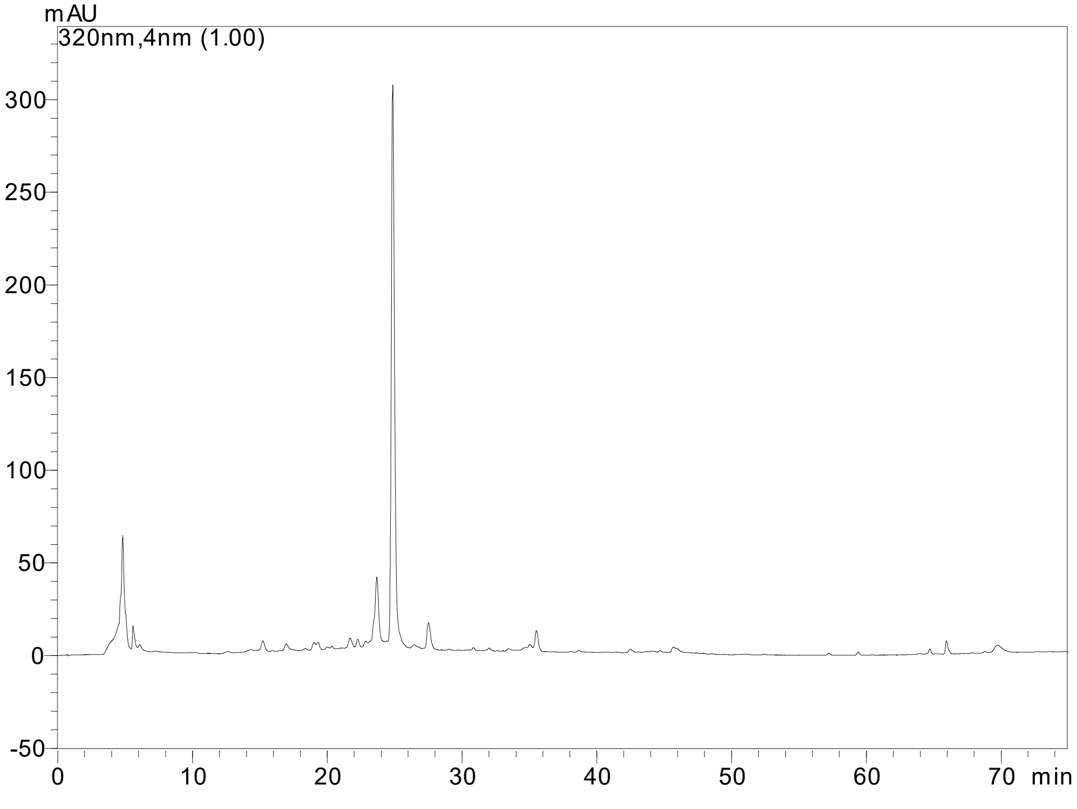

Supplementary Figure 10: HPL-chromatogram of cucumber peels recorded during LC-HR MS analysis. Cucumber was stored twelve days in the dark and the peel was extracted as described in the methods section. (Detection at 320 nm)

Source	type	Retention time LC-HR MS	Retention time LC	R1	R2	R3	R4	published PleB structures	published PxB structures
iceberg lettuce	PleB	14,45	20,7	O-Glc	CH_3	H	CH(OH)-CH_2OH	*Zm*-PleB-1, *Tc*-PleB-1, *Co*-PleB-1, *Ug*-PleB-27, *Pd*-PleB-35	
	PxB	14,9	21,4	O-Glc	CH_3	H	CH(OH)-CH_2OH		*Tc*-PxB-1, *Ep*-PxB-2
	PxB		19,5	O-(6'-OMal)Glc	CH_3	H	CH(OH)-CH_2OH		*Ep*-PxB-1
	PleB	9,58	18,7	O-(6'-OMal)Glc	CH_3	H	CH(OH)-CH_2OH		

Source	type	Retention time LC-HR MS	Retention time LC	R1	R2	R3	R4	published PleB structures	published PxB structures
Cucumber	PleB	13,73	21,3	O-Glc	CH_3	H	CH(OH)-CH_2OH	*Zm*-PleB-1, *Tc*-PleB-1, *Co*-PleB-1, *Ug*-PleB-27, *Pd*-PleB-35	
	PleB	15,9	22,6	OH	CH_3	H	CH(OH)-CH_2OH	*So*-PleB-2, *Mc*-PleB-42, *Pd*-PleB-40, *Cl*-PleB-2, *Ej*-PleB-1	
	PxB	19,65	23,3	OH	CH_3	H	CH(OH)-CH_2OH		*Ep*-PxB-3

Supplementary Table 1: Identified phyllobillins by UV Vis online spectra and LC-HR MS analysis. (R^{1-4} see Scheme 1; *Zm=Zea mays*, *Tc=Tilia cordata*, *Co=Cydonia oblonga*, *Ug=Ulmus glabra*, *Pd=Prunus x domestica*, *Ep=Echinacea purpurea*, *So=spinacia oleracea*, *Mc=Musa cavendish*, *Ej=Eribotrya japonica*)

Consumer Study

1. Tables and Figures

	Total	Control	Safety	Phyllobilin
**Gender in %**				
Female	50.6	49.0	50.5	52.2
Male	49.3	51.0	49.5	47.5
Other	0.1	0.0	0.0	0.3
**Age in %**				
18 – 22	4.9	5.2	5.1	4.4
23 – 35	19.4	19.1	18.1	21.2
36 – 55	37.0	37.3	38.8	34.7
56 +	38.7	38.4	38.0	39.7
**Education in %**				
No education completed (yet)	1.7	1.7	1.6	1.7
Primary education	32.4	31.0	32.3	33.8
Secondary education	32.0	31.9	32.1	32.1
University entrance qualification	33.9	35.5	34.0	32.4
**Household Size in %**				
1	33.1	33.8	32.2	33.3
2	39.8	37.3	38.6	43.6
3	14.0	15.3	15.2	11.4
4 +	13.1	13.6	14.1	11.7
**Income in %**				
Less than 1500€	27.4	24.6	29.7	27.9
1500 – 2500€	28.5	30.3	24.1	31.1
2500 – 3500€	19.5	17.6	22.7	18.0
3500 – 4500€	14.8	16.5	14.0	14.0
4500 – 5500€	6.2	6.9	5.9	5.8
More than 5500€	3.6	4.0	3.6	3.2
**Region in %**				
Baden-Württemberg	12.6	12.8	13.3	11.7
Bavaria	15.0	13.9	16.2	15.0
Berlin	4.6	3.5	5.9	4.4
Brandenburg	3.0	4.4	2.4	2.2
Bremen	0.8	0.5	1.1	0.8
Hamburg	3.3	2.5	4.5	2.8
Hesse	7.3	7.1	6.9	7.8
Mecklenburg Western Pomerania	2.0	3.0	1.9	1.1
Lower Saxony	10.0	12.0	8.2	9.7
Northrhine-Westphalia	21.8	22.3	19.7	23.3
Rhineland Palatinate	4.1	3.8	4.3	4.2
Saarland	1.8	2.5	0.5	2.5
Saxony	5.5	4.6	6.9	5.0
Saxony-Anhalt	2.5	2.7	2.1	2.8
Schleswig-Holstein	2.7	2.2	2.7	3.3
Thuringia	3.0	2.2	3.5	3.3

Supplementary Table 2: Sample Description (Total and Conditions)
a

**Number of days on which subjects were willing to consume the lettuce (ANOVA):**						
	**N**	**Mean**	**SD**	**95%-CI**	**F**	**p**
**Control**	367	3.62	2.15	3.40; 3.84		
**Safety**	376	4.15	2.26	3.92; 4.38		
**Phyllobilin**	360	4.19	2.31	3.95; 4.43		
**Total**	1103	3.99	2.25	3.85; 4.12	7.270	0.001

b

**Significance of Mean Differences (MD) between Groups Post-hoc Test:**			
		**MD**	**p**
**Control**	**Safety**	-.53*	0.004
	**Phyllobilin**	-.56*	0.002
**Safety**	**Phyllobilin**	-.03	0.976

* The difference is significant on the 1% level

Supplementary Table 3: Main Effect Information Treatment. (**a**) Means, Standard Deviations (SD), 95%-Confidence Interval (CI) of the Mean and Results from One-way Analysis of Variance (ANOVA). (**b**) Post-hoc Test for the Main Effect (Tukey-HSD).

Day	Condition	N	Mean	SD	95%-CI	F	p
**1**	**Control**	367	1.59	0.80	1.51; 1.67		
	**Safety**	376	1.58	0.78	1.50;1.66		
	**Phyllobilin**	360	1.64	0.73	1.56; 1.71		
	**Total**	1103	1.60	0.77		0.588	0.555
**2**	**Control**	323	1.72	0.72	1.64; 1.80		
	**Safety**	337	1.63	0.66	1.56; 1.70		
	**Phyllobilin**	322	1.66	0.65	1.58; 1.73		
	**Total**	982	1.67	0.68		1.587	0.205
**3**	**Control**	302	2.04	0.71	1.96; 2.12		
	**Safety**	320	1.93	0.75	1.85; 2.01		
	**Phyllobilin**	307	1.92	0.65	1.85; 2.00		
	**Total**	929	1.96	0.70		2.894[a]	0.056
**4**	**Control**	266	2.35 (a)	0.78	2.26; 2.44		
	**Safety**	283	2.10 (b)	0.73	2.02; 2.19		
	**Phyllobilin**	278	2.10 (b)	0.76	2.02; 2.19		
	**Total**	827	2.18	0.76		9.217[a]	0.000
**5**	**Control**	186	2.47 (a)	0.72	2.37; 2.58		
	**Safety**	237	2.24 (b)	0.75	2.15; 2.34		
	**Phyllobilin**	216	2.11 (b)	0.76	2.01; 2.21		
	**Total**	639	2.27	0.76		11.868	0.000
**6**	**Control**	122	2.59 (a)	0.74	2.46; 2.72		
	**Safety**	186	2.40 (a)	0.80	2.28; 2.51		
	**Phyllobilin**	167	2.11 (b)	0.80	1.99; 2.24		
	**Total**	475	2.35	0.81		13.612	0.000
**7**	**Control**	78	2.59 (a)	0.71	2.43; 2.75		
	**Safety**	113	2.35 (a)	0.80	2.20; 2.50		
	**Phyllobilin**	125	2.03 (b)	0.79	1.89; 2.17		
	**Total**	316	2.28	0.81		13.097	0.000

Supplementary Table 4: Health Perception of Lettuce by Group on Days 1 to 7: Means, Standard Deviations (SD), 95%-Confidence Interval (CI) of the Mean and Results from One-way ANOVA incl. Post-hoc Test. Scale: 1 = very healthy to 5 = very unhealthy.

[a] Welch's F used for data that violated the assumption of homogeneity of variance

(a) (b) – different letters indicate significant mean differences ($p<.05$) between the respective groups, evaluated by Tukey-HSD Post-hoc Test (for data that violated the assumption of homogeneity of variance Games Howell Post-hoc Test was used).

Day	Condition	N	Mean	SD	95%-CI	F	p
**1**	**Control**	367	1.59	0.81	1.51; 1.68		
	**Safety**	376	1.60	0.83	1.51; 1.68		
	**Phyllobilin**	360	1.61	0.81	1.52; 1.69		
	**Total**	1103	1.60	0.81	1.55; 1.65	0.029	0.971
**2**	**Control**	323	1.65	0.76	1.57; 1.74		
	**Safety**	337	1.55	0.70	1.48; 1.63		
	**Phyllobilin**	322	1.57	0.70	1.49; 1.65		
	**Total**	982	1.59	0.72	1.55; 1.64	1.815	0.163
**3**	**Control**	302	1.91	0.81	1.82; 2.00		
	**Safety**	320	1.78	0.78	1.69; 1.86		
	**Phyllobilin**	307	1.77	0.75	1.69; 1.86		
	**Total**	929	1.82	0.78	1.77; 1.87	3.055	0.048
**4**	**Control**	266	2.24 (a)	0.89	2.13; 2.35		
	**Safety**	283	1.94 (b)	0.79	1.84; 2.03		
	**Phyllobilin**	278	2.03 (b)	0.85	1.93; 2.13		
	**Total**	827	2.07	0.85	2.01; 2.12	9.082[a]	0.000
**5**	**Control**	186	2.31 (a)	0.87	2.18; 2.43		
	**Safety**	237	2.14	0.79	2.04; 2.24		
	**Phyllobilin**	216	2.04 (b)	0.82	1.93; 2.15		
	**Total**	639	2.15	0.83	2.09; 2.22	5.092[a]	0.007
**6**	**Control**	122	2.43 (a)	0.85	2.27; 2.58		
	**Safety**	186	2.32 (a)	0.85	2.20; 2.45		
	**Phyllobilin**	167	2.06 (b)	0.84	1.93; 2.19		
	**Total**	475	2.26	0.86	2.18; 2.33	7.531	0.001
**7**	**Control**	78	2.38 (a)	0.76	2.21; 2.56		
	**Safety**	113	2.26	0.87	2.09; 2.42		
	**Phyllobilin**	125	2.02 (b)	0.89	1.87; 2.18		
	**Total**	316	2.20	0.87	2.10; 2.29	4.709	0.010

Supplementary Table 5: Safety Perception of Lettuce by Group on Days 1 to 7: Means, Standard Deviations (SD), 95%-Confidence Interval (CI) of the Mean and Results from One-way ANOVA incl. Post-hoc Test. Scale: 1 = very safe to 5 = very unsafe.

[a] Welch's F used for data that violated the assumption of homogeneity of variance

(a) (b) – different letters indicate significant mean differences (p<.05) between the respective groups, evaluated by Tukey-HSD Post-hoc Test (for data that violated the assumption of homogeneity of variance Games Howell Post-hoc Test was used).

a

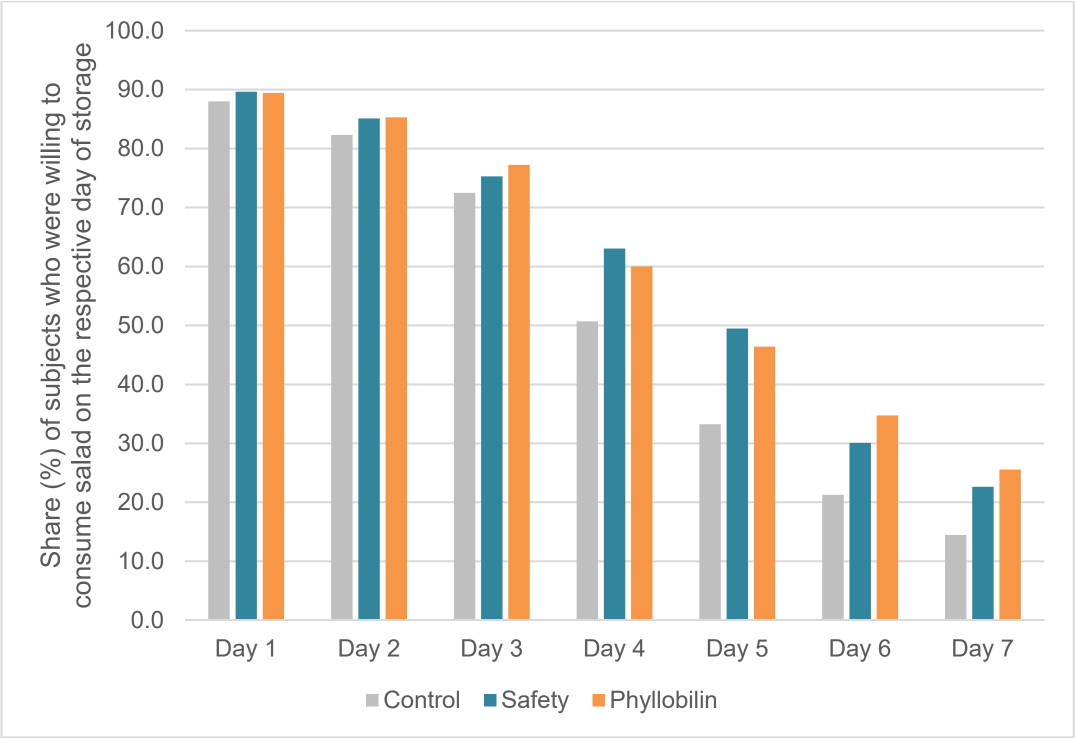
Share (%) of subjects who were willing to consume salad on the respective day of storage
100.0
90.0
80.0
70.0
60.0
50.0
40.0
30.0
20.0
10.0
0.0
Day 1
Day 2
Day 3
Day 4
Day 5
Day 6
Day 7
Control
Safety
Phyllobilin

b

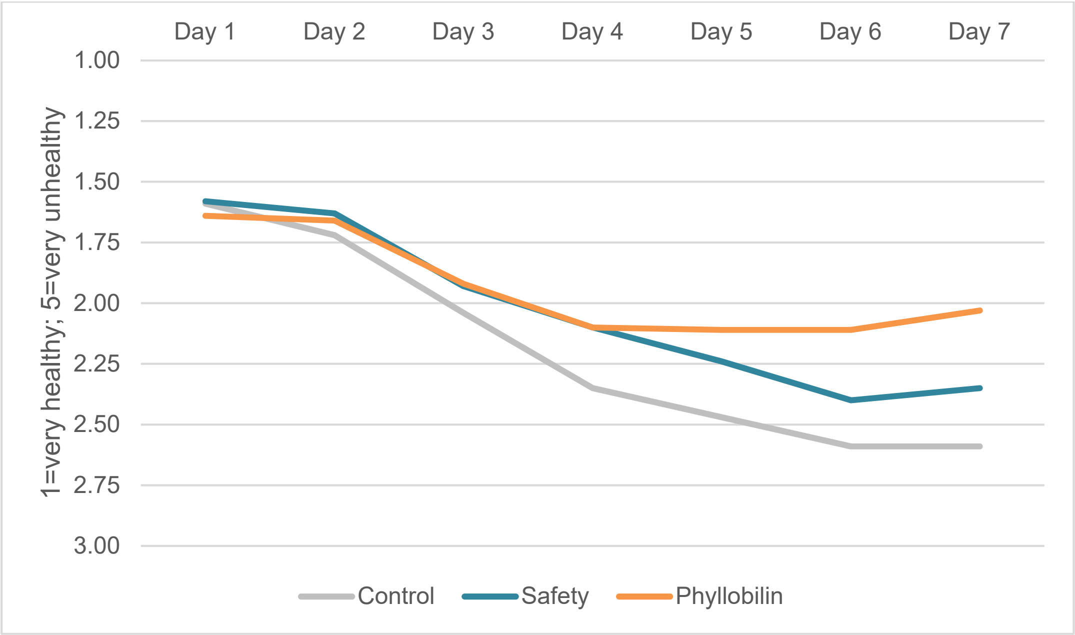
Day 1
Day 2
Day 3
Day 4
Day 5
Day 6
Day 7
1=very healthy; 5=very unhealthy
1.00
1.25
1.50
1.75
2.00
2.25
2.50
2.75
3.00
Control
Safety
Phyllobilin

c

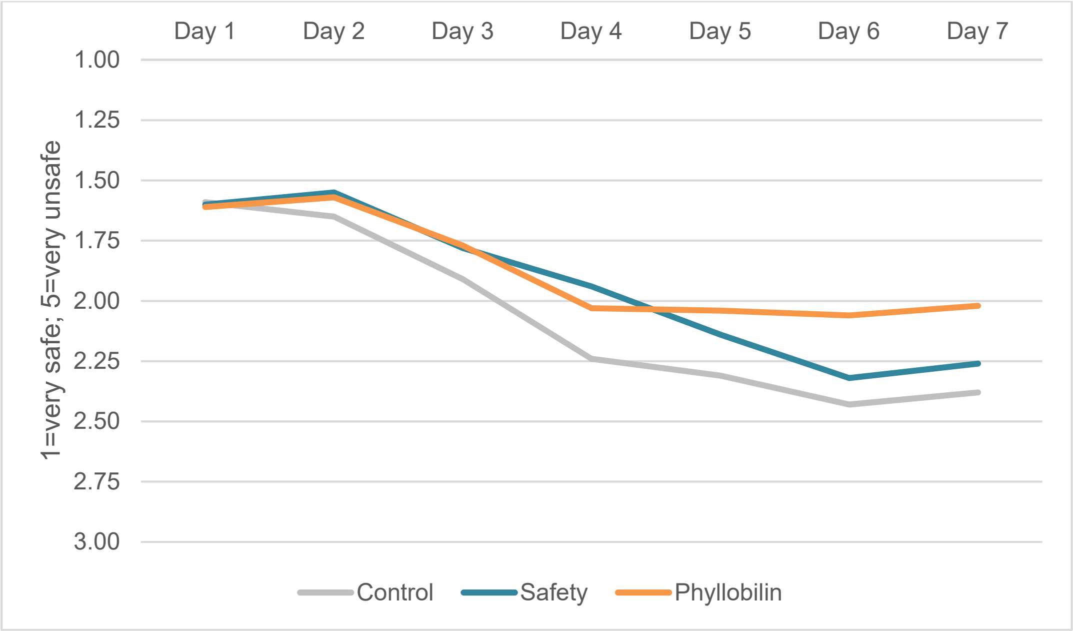

Supplementary Figure 11: Graphical Depiction of Results for Phyllobilin, Safety and Control Condition. (**a**) Share (%) of subjects in the phyllobilin, safety and control condition who stated to be willing to consume the lettuce on days 1 to 7 of storage. (**b**) Health perception of the lettuce (on 5-point Likert scale) on days 1 to 7 of storage by group. (**c**) Safety perception of the lettuce (on a 5-point Likert scale) on days 1 to 7 of storage by group.

2. Picture Material of the Lettuce shown to Subjects (one picture for each day)

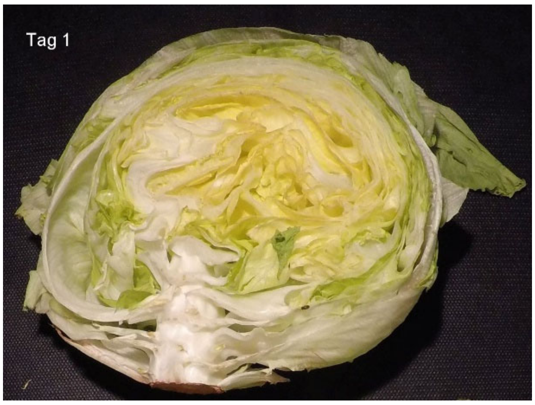

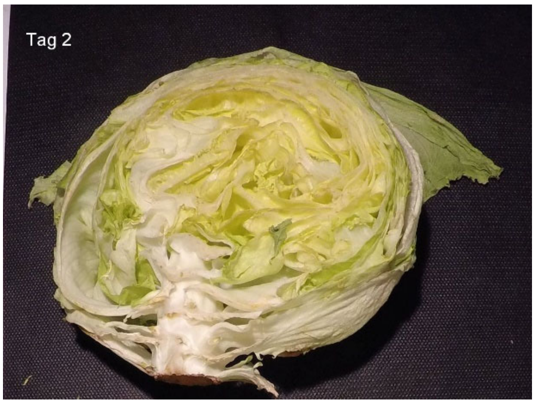

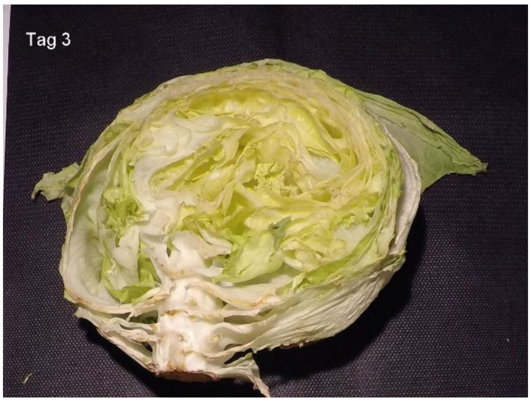

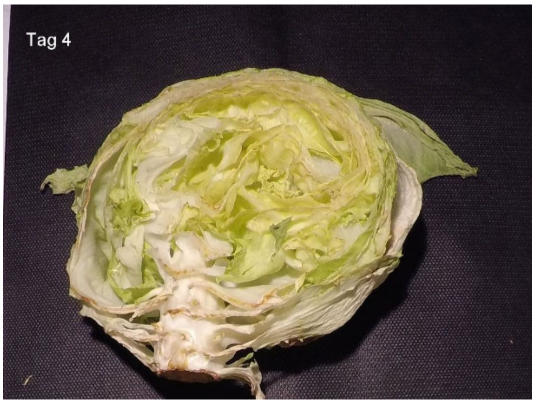

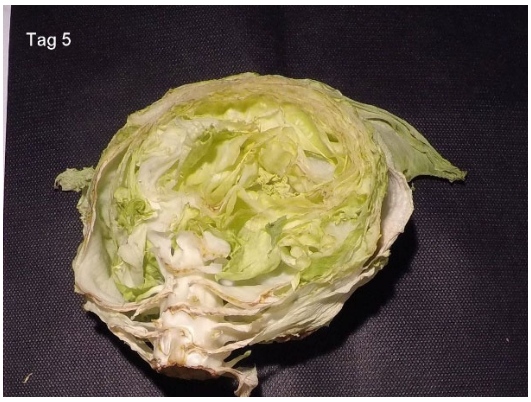

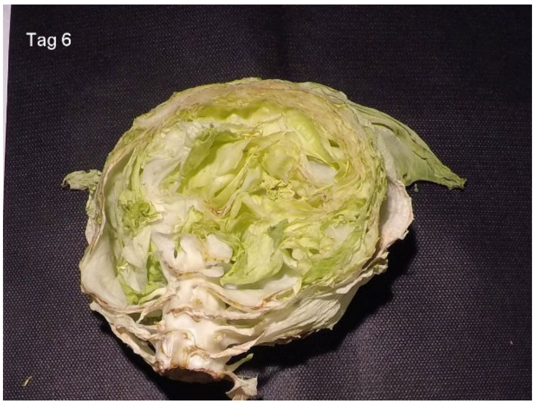

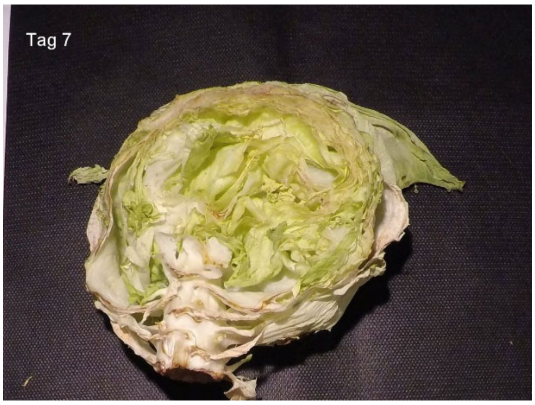

3. **Extract from the Consumer Questionnaire: Questions regarding willingness to consume lettuce, health perception, and safety perception.**

Example Day 1

(Questions were repeated for each day, until subject stated to no longer be willing to eat the lettuce)

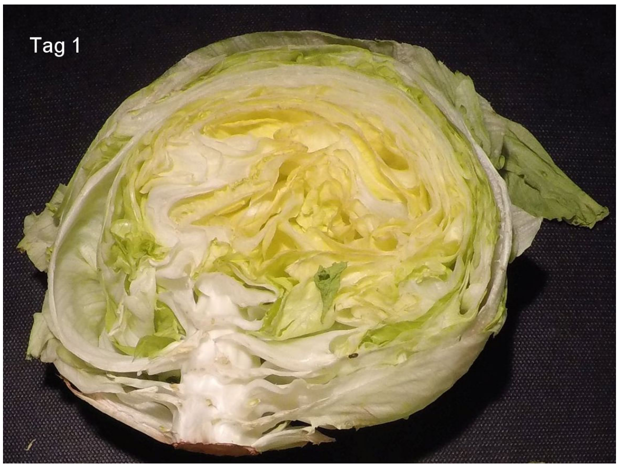

What do you think – how healthy or unhealthy is this lettuce?

- o Very healthy
- o Rather healthy
- o Neither
- o Rather unhealthy
- o Very unhealthy

In terms of potential health risks, how safe or unsafe would you consider it to eat this lettuce?

- o Very safe
- o Rather safe
- o Neither
- o Rather unsafe
- o Very unsafe

Would you still eat the lettuce?

- o Yes
- o No
- o Don’t know

4. Information Treatments provided to subjects before they answered the lettuce questions (see F.). Above the info, there was a time-lapse video of the lettuce aging over 7 days.

4.1 Control Treatment

The video (no sound) uses the example of lettuce to show how foods may change with ripening and aging. At the top left you can see the respective day and time.

As green foods, such as lettuce, age, the green pigment (chlorophyll) in the plant cells is broken down.

Chlorophylls are color pigments that are responsible for the absorption of light during photosynthesis and thus for the conversion of light energy into chemical energy. In this process, carbon dioxide and water are turned into oxygen and sugar. Chlorophylls are therefore essential components of one of the most important processes on earth, without which higher life would not be possible.

The amount of chlorophyll in the plant cells changes with the aging process. The result can be seen in the video: With the breakdown of chlorophyll, the lettuce becomes less and less green over time and takes on a brownish color.

4.2 Phyllobilin Treatment

The video (no sound) uses the example of lettuce to show how foods may change with ripening and aging. At the top left you can see the respective day and time.

As green foods, such as lettuce, age, the green pigment (chlorophyll) in the plant cells is broken down. As you can see in the video, the lettuce becomes less and less green over time and takes on a brownish color.

However, other chemical substances are produced during the breakdown of the green pigment. Among them are so-called phyllobilins. Phyllobilins are powerful antioxidants that protect our cells from oxidative stress. Oxidative stress can occur, for example, through inflammatory processes or environmental influences such as UV radiation or exhaust gases and contribute to the development of many different diseases. Antioxidants help our bodies deal with this stress.

Since phyllobilins arise with the aging of green vegetables, the content of phyllobilins is higher the older e.g. a cucumber or a salad is. For example, an iceberg lettuce stored for seven days contains up to 1000 times more phyllobilins than a freshly purchased one. So we take in certain amounts of phyllobilins with our diet every day. However, by eating vegetables that are no longer quite as fresh, we can significantly increase these quantities.

Studies of the lettuce shown in the video in the laboratory have also shown that the bacterial load does not increase between day 1 and day 7. Slightly brown and wilted lettuce is therefore not always bad, but can usually be consumed without hesitation and even benefit our health due to the increased phyllobilin content.

4.3 Safety Treatment

The video (no sound) uses the example of lettuce to show how foods may change with ripening and aging. At the top left you can see the respective day and time.

As green foods, such as lettuce, age, the green pigment (chlorophyll) in the plant cells is broken down. As you can see in the video, the lettuce becomes less and less green over time and takes on a brownish color. The brownish color and the wilting of the lead to the salad appearing less appetizing and often makes us worry whether we can still eat it without hesitation.

To answer this question, the microbial contamination of the lettuce shown in the video was measured in the laboratory. The results show that the bacterial load does not increase between day 1 and day 7. Slightly brown and wilted lettuce is therefore not necessarily bad and dangerous to eat, but can usually be eaten without hesitation.

Tip: rely on your senses! For example, if the salad is moldy or smells musty, it is better to throw it away. If this is not the case, you can still eat it!

Supplementary References

1 Kräutler, B. Breakdown of Chlorophyll in Higher Plants—Phyllobilins as Abundant, Yet Hardly Visible Signs of Ripening, Senescence, and Cell Death. *Angew. Chem. Int. Ed.* **55**, 4882-4907 (2016).

2 Christ, B., Hauenstein, M. & Hörtensteiner, S. A liquid chromatography–mass spectrometry platform for the analysis of phyllobilins, the major degradation products of chlorophyll in Arabidopsis thaliana. *Plant J.* **88**, 505-518 (2016).

3 Müller, T., Vergeiner, S. & Kräutler, B. Structure elucidation of chlorophyll catabolites (phyllobilins) by ESI-mass spectrometry—Pseudo-molecular ions and fragmentation analysis of a nonfluorescent chlorophyll catabolite (NCC). *Int. J. Mass Spectrom.* **365-366**, 48-55 (2014).

4 Pružinska, A., Tanner, G., Anders, I., Roca, M. & Hörtensteiner, S. Chlorophyll breakdown: Pheophorbide *a* oxygenase is a Rieske-type iron-sulfur protein, encoded by the *accelerated cell death 1* gene. *Proc. Natl. Acad. Sci. USA* **100**, 15259-15264 (2003).

5 Strain, H. H., Cope, B. T. & Svec, W. A. in *Methods in Enzymology* Vol. 23 452-476 (Academic Press, 1971).
